# Editorial: Evidenced based medical care of hospitalized children with local adaptations in low-resource settings

**DOI:** 10.3389/fped.2023.1198673

**Published:** 2023-07-26

**Authors:** C. R. Howard, D. A. Gbadero, T. M. Slusher, F. Bode-Thomas

**Affiliations:** ^1^ Department of Pediatrics, Global Pediatrics Program, University of Minnesota, Minneapolis, MN, United States; ^2^ Department of Paediatrics, Bowen University, Iwo, Nigeria; ^3^ Department of Paediatrics, Bowen University Teaching Hospital, Ogbomoso, Nigeria; ^4^ Department of Paediatrics, Hennepin Healthcare, Minneapolis, MN, United States; ^5^ Department of Paediatrics, University of Jos, Jos, Nigeria; ^6^ Department of Paediatrics, Jos University Teaching Hospital, Jos, Nigeria

**Keywords:** low-resource, hospitalized, children, adaptations, evidence-based

**Editorial on the Research Topic** Evidenced based medical care of hospitalized children with local adaptations in low-resource settings

The series of articles recently published in Frontiers in Pediatrics explored *evidence-based medical care of hospitalized children with local adaptations in low-resource settings*. Successful implementation of life saving medical care for children in local settings was described by Satrom et al. and Wu et al.. Gaps in implementation of diagnostic and therapeutic modalities to prevent childhood morbidity and mortality were clearly illustrated in the studies from Han et al., Menalu et al., Villanueva-Uy et al., and Ba-alwi et al.. We were encouraged to see the innovations captured by recent advances in the identification of neonatal jaundice and the prevention of acute bilirubin encephalopathy (Satrom et al., Villanueva-Uy et al.); and ingenuity, well described by Wu et al. as they highlighted new methods to breathe for infants and children who would otherwise die due to acute respiratory failure secondary to prematurity or lower respiratory tract infections.

The pivotal role of education was emphasized including maternal and village health workers’ education in the recognition of neonatal jaundice and neurological dysfunction (Satrom et al.); the creative adaptation of virtual education across continents reported by Le Pichon to train physicians who do not have access to sub-specialty expertise in their own locale (Le Pichon et al.); and partnership was beautifully illustrated by Fant et al. as they reported on the Maseno University-Northwestern University simulation-based medical education collaborative. Household education and prevention were also emphasized in the study of the epidemiology of burns in central China by Han et al.. Neonatal survival studies from Ethiopia and Tanzania highlighted the ongoing challenge of improving newborn survival (Menalu et al., Ba-alwi et al.). Both captured the need for early intervention and treatment.

Prevention is the focus of our specialty as pediatricians and vaccines are our most powerful weapon to prevent infectious diseases and their morbidity and mortality as in the case of the multisystem inflammatory syndrome of COVID. Douangboupha et al. call for expanded access to SARS-CoV-2 vaccine for children in addition to equitable care for children no matter where they live especially in resource limited settings.

The common thread through all these reports from Asia and Africa is implementation of what we already know works to save the lives of children no matter where they live. There are many barriers to this goal. One challenge is assuring that every child has access to prevention, which is often as simple as maternal education about how to child proof the home to prevent burns and as complicated as barriers to available, accessible, affordable, and effective vaccines. State of the art laboratories are woefully limited in many parts of the world. Availability, accessibility, and affordability of life saving medicines are a barrier to implementation of evidence-based treatment, insulin being a prime example throughout the world.

Authors from Asia, Africa and North America are represented in these studies/publications which remind us of how creative, innovative, ingenious, and relevant we can be in pediatric medicine across the globe. We are also reminded of the inequities in parental education, sub- specialty training, laboratory diagnosis, and medical care. Implementation science is the frontier we as pediatricians need to fully engage across multiple disciplines including political and economic disciplines with innovation and determination in ethical and sustainable partnerships if we are to close every gap identified in these studies. Filling these gaps requires the interdisciplinary expertise of individuals from resource limited areas of a country or continent to be fully funded and in leadership roles to direct implementation research with the goal of adapting the wealth of evidence based medical care for hospitalized children to the local community of every child. Working together in truly equitable, ethically sound partnerships across the globe we can tackle the challenges to health and well-being our children face as demonstrated in this series of articles.

